# New sexually transmitted HIV infections from 2016 to 2050 in Guangdong Province, China: a study based on a dynamic compartmental model

**DOI:** 10.1186/s12889-024-18735-z

**Published:** 2024-05-14

**Authors:** Rong Ye, Yingsi Lai, Jing Gu

**Affiliations:** 1 Department of Medical Statistics, School of Public Health, Sun Yat-sen University, No. 74 Zhongshan 2nd Road, Guangzhou, 510080 China; 2 Sun Yat-sen Global Health institute, Sun Yat-sen University, Guangzhou, China; 3 Research Center of Health Informatics, Sun Yat-sen University, Guangzhou, China

**Keywords:** HIV, Sexual transmission routes, Men who have sex with men and women, General population, Guangdong Province, People’s Republic of China

## Abstract

**Background:**

In Guangdong Province, China, there is lack of information on the HIV epidemic among high-risk groups and the general population, particularly in relation to sexual transmission, which is a predominant route. The new HIV infections each year is also uncertain owing to HIV transmission from men who have sex with men (MSM) to women, as a substantial proportion of MSM also have female sexual partnerships to comply with social demands in China.

**Methods:**

A deterministic compartmental model was developed to predict new HIV infections in four risk groups, including heterosexual men and women and low- and high-risk MSM, in Guangdong Province from 2016 to 2050, considering HIV transmission from MSM to women. The new HIV infections and its 95% credible interval (CrI) were predicted. An adaptive sequential Monte Carlo method for approximate Bayesian computation (ABC-SMC) was used to estimate the unknown parameter, a mixing index. We calibrated our results based on new HIV diagnoses and proportions of late diagnoses. The Morris and Sobol methods were applied in the sensitivity analysis.

**Results:**

New HIV infections increased during and 2 years after the COVID-19 pandemic, then declined until 2050. New infections rose from 8,828 [95% credible interval (CrI): 6,435–10,451] in 2016 to 9,652 (95% CrI: 7,027–11,434) in 2019, peaking at 11,152 (95% CrI: 8,337–13,062) in 2024 before declining to 7,084 (95% CrI: 5,165–8,385) in 2035 and 4,849 (95% CrI: 3,524–5,747) in 2050. Women accounted for approximately 25.0% of new HIV infections, MSM accounted for 40.0% (approximately 55.0% of men), and high-risk MSM accounted for approximately 25.0% of the total. The ABC-SMC mixing index was 0.504 (95% CrI: 0.239–0.894).

**Conclusions:**

Given that new HIV infections and the proportion of women were relatively high in our calibrated model, to some extent, the HIV epidemic in Guangdong Province remains serious, and services for HIV prevention and control are urgently needed to return to the levels before the COVID-19 epidemic, especially in promoting condom-based safe sex and increasing awareness of HIV prevention to general population.

**Supplementary Information:**

The online version contains supplementary material available at 10.1186/s12889-024-18735-z.

## Background

HIV remains a worldwide public health threat, while the efforts for prevention and control efforts have made substantial progress, including reducing of HIV-related morbidity and mortality and increasing life expectancy and access to antiretroviral therapy. The number of people living with HIV was 38.4 million according to the Joint United Nations Programme on HIV/AIDS (UNAIDS) at the end of 2021 [1], and the number of new HIV infections was approximately 1.5 million [2]. The number of people living with HIV was estimated to be 1.25 million in China in 2018, with approximately 80,000 new infections that year [3]. As of 2022, the number of people living with HIV was over 80 thousand in Guangdong Province, which ranked fourth out of 31 provinces [4].

Guangdong Province is located in southern China and has a relatively advanced economy and a higher proportion of sexually active individuals [5]. The number of new HIV diagnoses each year is high and increasing owing to the complex mode of sexual transmission, which has been the main route since 2009 [6], accounting for 52.4%, and increasing continually to 90.3% in 2014. However, the number of new HIV infections predicted in Guangdong Province has not been reported in published peer-reviewed articles. It is important to predict the number of new HIV infections in provinces where incidences of HIV may be different for prevention and control planning, especially since heterosexual transmission is the dominant transmission mode in China.

HIV is increasingly difficult to prevent and control due to intricate modes of sexual transmission in China, including heterosexual transmission from heterosexual men or men who have sex with men (MSM) in heterosexual acts and homosexual transmission. It is observed that HIV began to spread to the general population from key populations in 2007 [4, 7]. Furthermore, impacted by the traditional culture including marital pressures and filial expectations [8, 9], the mode of sexual transmission makes the HIV epidemic complex and diverse due to mixed sexual acts in marital or nonmarital partnerships, commercial or noncommercial partnerships, heterosexual or homosexual partnerships, etc [10].

There are different methods to estimate the number of new HIV infections including mathematical models such as compartmental models and the workbook method; however, a compartmental model is often used to estimate the number of infections [11], which is also recommended by UNAIDS [12–14]. Dynamic compartmental models that go beyond the workbook method [15] in long-term prediction are also classical mathematical models of infectious disease transmission, but there are two considerable challenges in developing a compartmental model to predict new HIV infections, including defining risk groups related to MSM depending on the characteristics of a local epidemic and defining a criterion or a reference for model calibration in addition to parameter estimation.

One definition of MSM considers sex of their partners in sexual acts in addition to their number of sexual partnerships in predictive models of new HIV infections; some MSM may have female sexual partnerships in some countries and are also known as men who have sex with men and women (MSMW). The marriage rate among Chinese MSM ranges from 31.2 to 70% [16–19] and is even higher during the lifetime [20], and the number of wives (tongqi) of MSM is between 1 million and 16 million [21]. However, MSM included only two subgroups depending on their number of sexual partnerships in previous compartmental models [22, 23]. This was inconsistent with the characteristics of an epidemic in these countries as it ignored the risk of HIV transmission [24–26], which was higher among MSMW than among MSM only [27], even though MSM are often classified into two subgroups that make interpretability insufficient between the number of predictions and the number of case reports [3]. MSMW may transmit HIV [26, 28]. With the HIV prevalence among MSM increasing, the HIV incidence among their female partners increased by 5.3 times from 2002 to 2010 [29] because MSM failed to disclose their sexual orientation and HIV seropositivity to their wives or female sexual partnerships to satisfy the social and familial expectations [28, 30] in general, thus reducing the awareness of HIV prevention [31] during heterosexual acts with lower condom use than during homosexual acts due to reproductive purposes [18, 32] or other reasons [33–35].

Another challenge is how to determine a criterion for model calibration for a compartmental model. Given that the problem of late diagnosis is prevalent worldwide [36–39] and continues to be prevalent [40], model calibration must also consider the proportion in addition to the number of new HIV diagnoses each year, which was often employed to calibrate owing to a lack of other data. The number predicted must be almost higher than the number of new HIV diagnoses each year in most countries due to late diagnoses [37, 38, 41, 42], whose proportion may increase owing to the impacts of the COVID-19 pandemic [43]; otherwise, the predicted number will be underestimated to some extent. Hence, it is critical to determine the proportion of people diagnosed with HIV infections for model calibration, and the sources should be reasonable and credible and include published studies, AIDS expert interviews or internal estimations from the Centers for Disease Control and Prevention (CDC). This study aimed to predict the number of new HIV infections by developing a compartmental model for the population aged 15 and over considering two sexual transmission routes to further understand the HIV epidemic. Model calibration included the proportion of late diagnoses in addition to the number of new HIV diagnoses each year (almost equal to the number of new cases reported from medical and health institutions at all levels in China because of compulsory guidelines mandated by the National Notifiable Disease Reporting System).

## Methods

We developed a deterministic compartmental model with four states to predict the HIV epidemic in the population aged 15 and over in Guangdong Province from 2016 to 2050. The model predicted the number of new HIV infections and its 95% credible interval (CrI). We used the Morris and Sobol methods to analyse the sensitivity of the model parameters. The predicted number was calibrated by comparing it with the number of yearly new HIV diagnoses (almost equal to the number of new cases reported in China) and the potential proportion of late diagnoses reported by AIDS experts in the Guangdong Provincial CDC or published peer-reviewed articles [37]. The model structure, population size of risk groups, model parameters, sensitivity analysis and model calibration are as follows.

### Model structure

**Fig. 1:**
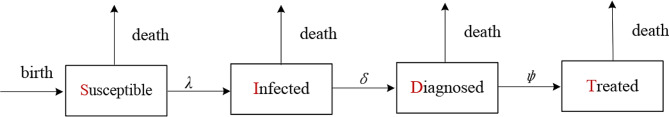
A schematic diagram of the compartmental model of HIV transmission

Considering births and deaths, the basic structure of a compartmental model with four states is shown in Fig. 1. Four ordinary differential equations describing the states are shown in formulas (1)–(4), denoting four states of the HIV epidemic, four risk groups, and two sexual transmission routes among MSM. The four states were S (susceptible), I (infected), D (diagnosed), and T (treated); the infectivity of people in the I and D states and the antiretroviral treatment failure of those in the T state were evaluated. We defined MSM as the high-risk MSM who satisfied at least one of two conditions [22, 23]: (1) more than 10 sexual partners over the past six months [44] and (2) rates of inconsistent condom use over the past six months more than 50.0%, which were approximately 50.0% in Guangdong Province [45]; otherwise, they were classified as low-risk MSM. The four risk groups were heterosexual men, heterosexual women, low-risk MSM, and high-risk MSM. The two sexual transmission routes were heterosexual and homosexual. The probability of HIV acquisition is denoted as $\lambda \left(t\right)$ in the S population per year. The *I*(*t*) is denoted as $\lambda \left(t\left)S\right(t\right)$. The number of people engaging in potentially high-risk sexual acts per year is denoted as $N\left(t\right)=S\left(t\right)+I\left(t\right)$, with *I* (*t*) including *D* (*t*) and *T* (*t*). ${\upzeta }\left(t\right)$ is the entry rate per year, which is the sum of the natural birth rate and natural death rate. $d\left(S,I,D,T\right)$ is the death rate in the states for the risk groups.

1 $$\frac{{{X_{j,1,i}}}}{{dt}} = \sum\limits_{j = 1}^4 {{\zeta _j}} {X_{j,1,i}} - \left( {\sum\limits_{k = {\text{2,3}},{4^\prime }} {\lambda _{i,k}^j} \left( t \right)} \right){X_{j,1,i}} - {d_{j,1}}{X_{j,1,i}}$$

2 $$\frac{{X}_{j,2,i}}{dt}=\left(\sum _{k=\text{2,3},{4}^{{\prime }}}{\lambda }_{i,k}^{j}\left(t\right)\right){X}_{j,1,i}-{d}_{j,2}{X}_{j,2,i}$$

3 $$\frac{{X}_{j,3,i}}{dt}={\delta }_{j}{X}_{j,2,i}-{d}_{j,3}{X}_{j,3,i}$$

4 $$\frac{{X}_{j,4,i}}{dt}={\psi }_{j}{X}_{j,3,i}-{d}_{j,4}{X}_{j,4,i}$$

$$j=\text{1,2},\text{3,4}, i=1, 2$$

j denotes the four risk groups, *j*$=\text{1,2},\dots ,4$, 1 indicates heterosexual men, 2 heterosexual women, 3 low-risk MSM, and 4 high-risk MSM. *i* denotes HIV sexual transmission routes, $i=\text{1,2}$, 1 indicates heterosexual sexual acts, and 2 indicates homosexual sexual acts. *k* denotes the four states, $k=\text{1,2},\dots ,4$’, where 1 is S, 2 is I, 3 is D, 4 is T, and $4$’ is treatment failure.

### Population sizes of the risk groups

The number of people in the risk groups, including heterosexual men, heterosexual women, low-risk and high-risk MSM, and low-risk and high-risk MSMW, whose risk groups among MSM or MSMW were classified based on the number of male sexual partners among MSM, were calculated on the basis of the population size and the proportion of people aged 15 and over by sex from then the 2020 Guangdong Statistical Yearbook, which includes data from 2019 and before [46]. Data from 2020 were used with the assumption that the population size has remained relatively steady. Presuming that the population size of low-risk MSM was equal to that of high-risk MSM [45], the proportion of MSM was 5.0% of all men aged 15 and over, and the proportion of heterosexual sexual acts among low- and high-risk MSM was 31.2% [16]. By excluding people already living with HIV, we estimated that the population sizes of heterosexual men, low- and high-risk MSM, and low- and high-risk MSMW were 47.89 million, 1.3 million, 1.3 million, 0.4 million, and 0.4 million, respectively. The population size of heterosexual women was 43.73 million, which excluded people living with HIV and a number of lesbians, whose proportion was presumed to be 5.0%, as the risk of HIV transmission is extremely rare in this group.

### Model parameters

Model parameters included three parts depending on whether they were known or unknown (unknown parameters: the probability of HIV acquisitions and a mixing index). The known parameters that we collected (see the Supplementary material) included demographic, behavioural, biological and epidemiological data, coming from peer-reviewed published articles, domestic government reports, AIDS expert interviews, and the viewpoints of key experts on World AIDS Day; these parameters were also employed in other studies and meta-analyses. Demographic parameters included the population sizes of the four groups of people aged 15 and over and the number of people who entered and left this population yearly because of natural population growth. Behavioural parameters included sexual partners, condom use rates and the effectiveness of condoms, the last two of which differed between heterosexual and homosexual sexual acts. Biological parameters included the probability of HIV transmission per sexual act and death rates in different states. Epidemiological parameters included HIV prevalence, rates of new diagnoses, and rates of antiretroviral therapy.

The first unknown parameter was the probability of HIV acquisition, which was a function of the probability of not acquiring HIV during a high-risk sexual act, the number of sexual acts, the number of sexual partners, condom use rates and the effectiveness of condoms. We presumed that HIV transmission in our model occurred only via sextual transmission, including heterosexual and homosexual acts, due to sexual transmission being the dominant route since 2009 in Guangdong Province; the rate of sexual transmission increased continually to 90.3% in 2014. We also presumed that heterosexual men engaged in only heterosexual sexual acts, and women were only infected by men who could also be MSMW. These men were indistinguishable from heterosexual men participating in heterosexual sexual acts due to the lack of a relevant compartment for heterosexual women in the compartmental model. The probability of acquiring HIV in a high-risk sexual act among MSM was calculated based on the total number of homosexual sexual acts, of which insertive and receptive anal sex were indistinguishable. People living with HIV who have experienced antiretroviral treatment failure can spread HIV, or they are not infectious and should achieve a suppressed viral load. The function and its calculation are detailed in the Appendix.

The last unknown parameter was a mixing index that we used approximate Bayesian computation (ABC) [47, 48] to estimate. The index was a randomized mixing level of opting for male or female sexual partners among MSM, also called the assortativity, ranging from 0 to 1 [49], where 0 denoted that MSM would only opt for male sexual partners and 1 denoted that MSM would opt for male or female sexual partners completely at random. The number of HIV infections approached to the number of HIV diagnoses as the randomized mixing level increased [49]. Finally, an adaptive sequential Monte Carlo (SMC) sampler [50] was adopted considering the cost of calculations, given that a value for the mixing index could not be acquired from an epidemiological research or published peer-reviewed studies, which only presented an unconvincing presumed value of 0.5 [23].

### Sensitivity analysis

Considering the cost of calculations, we combined a qualitative global method and a quantitative global method, the Morris and Sobol methods, to analyse the sensitivity of model parameters to the number of new HIV infections predicted in our model. The first step qualitatively determined the sensitive parameters via the Morris method [51] and then calculated the quantitative effect of those identified parameters on the number of new HIV infections predicted via the Sobol method [52]. The sensitivity analysis excluded the parameter of the mixing index because it was certainly sensitive. The outputs of the Sobol method included the total-order indices and the first-order indices on qualitatively identified parameters from the absolute values of elementary effects (|EEs|) of the Morris method. 

### Model calibration

The model calibration data included the following: the yearly new HIV diagnoses based on the system of case reports on government websites [53, 54] on World AIDS Day from 2016 to 2022; and the proportion of HIV diagnoses based on published peer-reviewed studies [41, 55, 56], the viewpoints of a national AIDS expert [3] or provincial AIDS expert interviews [57] from the Guangdong Provincial CDC. Moreover, the number of new HIV infections may have increased during the COVID-19 epidemic due to temporary disruptions in health services and changes in sexual risk acts, including interrupted antiretroviral treatment, reduced HIV testing, and a higher proportion of unprotected sexual acts. Considering the proportion of late diagnoses in Guangdong Province, the difference was measured by determining the relative ratios of the number of new predicted HIV infections divided by the number of new HIV diagnoses each year, which was deemed the “goodness of fit” of the model, and a value larger than 1.2 was applied as a measure of model calibration [37, 41, 58]. A 95% credible interval (CrI) of the posterior parameter from SMC-ABC was adopted to calculate the 95% CrI of the number of new HIV infections. Uncertainty intervals (UI) of it on heterosexual men and women were calculated by realistic scenarios of the minimum and maximum considering sensitivity parameters identified in our sensitivity analysis. To incorporate the potential impact of the COVID-19 pandemic, we made adjustments to the sensitive parameters in our model.

## Results

### Parameter estimation

The value of the mixing index estimated from ABC-SMC was 0.504 (95% CrI: 0.239, 0.894). The prior presumed distribution of the index was uniformly distributed [0, 1], the sampling sequences were set at 25, the total sample size of each sequence was 10,000, each sampling size was 10, and the effective sample size was also estimated.

### Sensitivity analysis

The |EEs| from the Morris method for the number of sexual partners, probabilities of acquiring HIV during high-risk sexual acts, the proportion of condom use and the population sizes of the four states were 3,811.9, 2,441.1, 136.0 and 440.4, respectively. The first three parameters were identified as sensitive parameters, as the population sizes were relatively stable. The Sobol method revealed the following ranking for the three parameters: the number of sexual partners, the probability of acquiring HIV, and the proportion of condom use (refer to Supplementary Fig. 1 for more information). The total-order indices for these parameters were calculated to be 0.73, 0.29, and 0.0009, respectively, while the first-order indices were found to be 0.70, 0.27, and 0.0007, respectively.

### Model calibration

The ratios between the predicted number of new HIV infections and the number of new diagnoses per year are also shown as relative ratios, with further adjustments of the sensitivity parameters on the basis of the unknown parameter estimation for the population that experienced sexual transmission in Guangdong Province. The relative ratios were all larger than 1.2 and were calculated according to the proportion of HIV diagnoses or late diagnoses in Guangdong Province (Table 1). The ratios during the COVID-19 pandemic were much larger than those before the pandemic.

**Table 1:** Model calibration results: Relative ratios larger than 1.2

Year	New HIV diagnoses	NHD*1.2	New HIV infections predicted	Relative ratios
2016	6,029	7,235	8,828 (6,435–10,451)	1.2
2017	6,201	7,442	9,223 (6,742–10,906)	1.2
2018	6,262	7,515	9,399 (6,787–11,172)	1.3
2019	6,076	7,292	9,652 (7,027–11,434)	1.3
2020	5,355	6,426	10,022 (7,531–11,712)	1.6
2021	6,131	7,358	10,527 (8,102–12,172)	1.4
2022	5,354	6,425	11,099 (8,494–12,867)	1.7

Notes: NHD refers to the number of new HIV diagnoses, which was almost equal to the number of new HIV cases reported from Jan. 1 to Oct. 31 of the year in which the data were released on World AIDS Day by the government of Guangdong Province. NHD*1.2, the number of new HIV diagnoses each year. Relative ratios denote the number of new predicted HIV infections divided by the number of new HIV diagnoses each year, which was larger than 1.2 in Guangdong; otherwise, the predicted number may have been underestimated because of the problem of late HIV diagnoses worldwide.

### Number of new infections

**Fig. 2:**
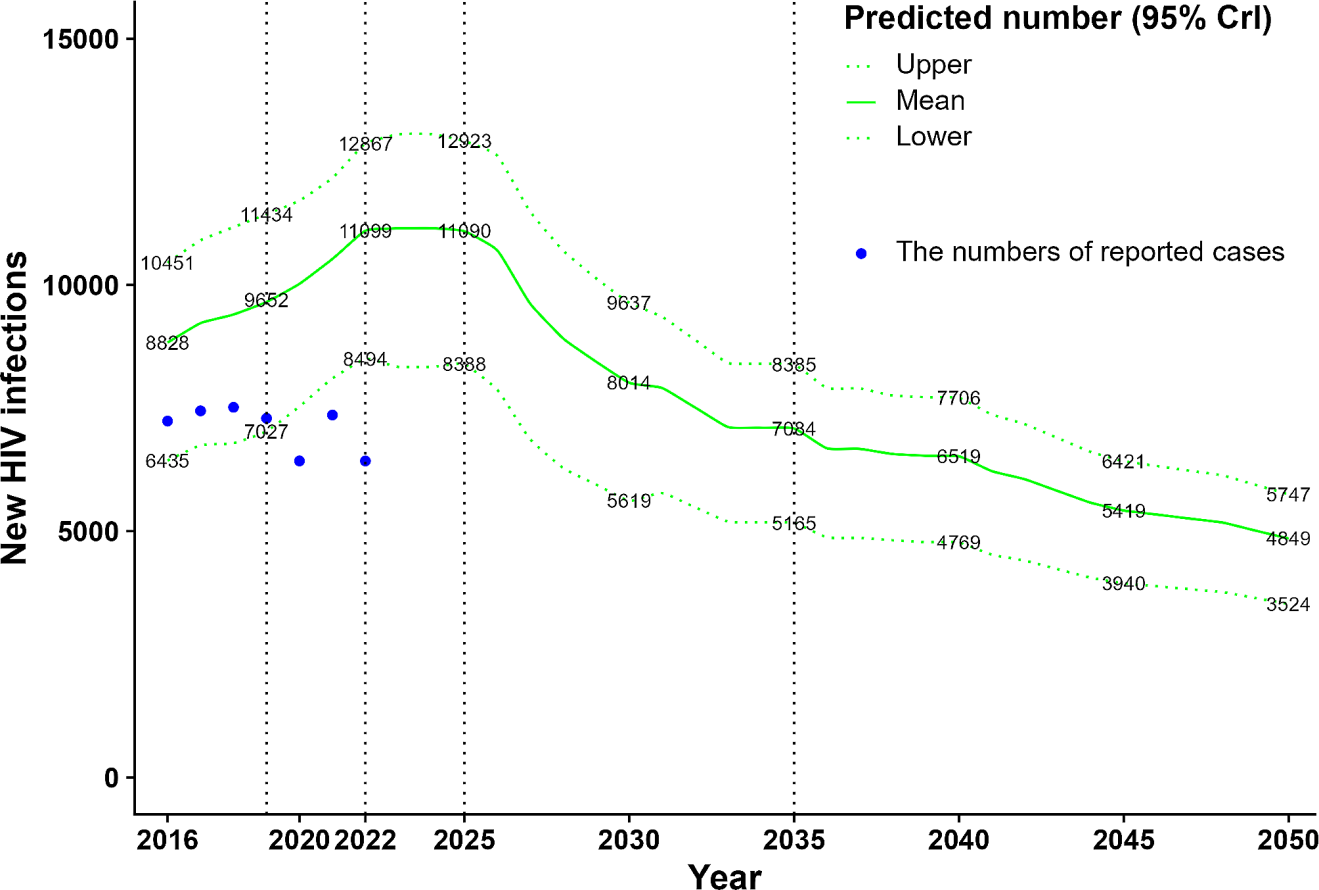
Predicted number (95% CrI) of new HIV infections in the population aged 15 and over from 2016 to 2050 in Guangdong Province, China

The number of new HIV infections in our compartmental model showed that the prediction period was comprehensively divided into five stages on the basis of changing trends (see Fig. 2), including before the COVID-19 epidemic (2016–2019), the COVID-19 epidemic (2020–2022), two or three years after the COVID-19 epidemic (2023–2025), the decade after the COVID-19 epidemic (2026–2035), and the period until 2050. The number of new HIV infections slightly increased from 2016 to 2019, sharply increased from 2020 to 2022, continuously increased from 2023 to 2025, declined yearly from 2026 to 2035, and remained at a stable lower level and slowly decreased until 2050. The number and proportion of new HIV infections predicted for the four risk groups were as follows.

The number of new HIV infections predicted each year in Guangdong Province was found to be 8,828 [95% credible interval (CrI): 6,435–10,451] in 2016, slightly increasing to 9,652 (95% CrI: 7,027–11,434) in 2019, continually jumping to 11,152 (95% CrI: 8,337–13,062) in 2024, beginning to decrease in 2025, then persistently declining to 7084 (95% CrI: 5,165–8,385) in 2035 and continuing to decline to 4,849 (95% CrI: 3,524–5,747) until 2050. The proportion of the predicted number of new HIV infections totally fell by approximately 45.0% between 2016 and 2050, as shown in Table 2. The increase in the predicted number of new HIV infections was much larger during the COVID-19 pandemic and the two years after the end of the pandemic, and then the number continuously declined until 2050. The changing tendency of the number of new HIV infections predicted for the four risk groups was in line with the changing tendency of the total number predicted each year (see Fig. 2).

**Table 2:** Predicted number of new HIV infections in four susceptible populations in Guangdong Province from 2016 to 2050

Year	*N*	Heterosexual males	Heterosexual females	LR_MSM	HR_MSM
2016	8,828 (6,435–10,451)	2,839 (2,237–3,243)	2,607 (2,359–3,016)	1,094 (329–1,612)	2,288 (660–3,393)
2017	9,223 (6,742–10,906)	3,165 (2,615–3,734)	2,538 (2,388–3,090)	1,199 (360–1,768)	2,321 (680–3,435)
2018	9,399 (6,787–11,172)	3,189 (2,698–3,842)	2,518 (2,267–2,967)	1,209 (359–1,786)	2,483 (721–3,680)
2019	9,652 (7,027–11,434)	3,118 (2,690–3,929)	2,835 (2,259–2,879)	1,209 (357–1,787)	2,490 (717–3,693)
2020	10,022 (7,531–11,712)	2,599 (2,407–3,619)	3,927 (3,775–4,983)	1,090 (320–1,612)	2,406 (685–3,574)
2021	10,527 (8,102–12,172)	3,154 (2,398–3,373)	3,944 (3,743–4,509)	1,256 (372–1,855)	2,173 (632–3,219)
2022	11,099 (8,494–12,867)	3,154 (2,332–3,502)	4,263 (3,868–5,244)	1,256 (373–1,856)	2,426 (705–3,595)
2023	11,149 (8,336–13,059)	2,991 (2,300–3,307)	4,159 (3,826–5,080)	1,433 (431–2,113)	2,566 (755–3,796)
2024	11,152 (8,337–13,062)	2,991 (2,388–3,422)	4,159 (3,619–4,859)	1,434 (431–2,115)	2,568 (756–3,798)
2025	11,090 (8,388–12,923)	2,901 (2,372–3,495)	4,346 (3,612–4,735)	1,424 (429–2,098)	2,419 (712–3,578)
2026	10,699 (7,868–12,621)	2,980 (2,511–3,779)	3,693 (3,289–5,358)	1,487 (448–2,193)	2,539 (747–3,755)
2027	9,618 (6,865–11,486)	2,959 (2,648–3,396)	2,744 (2,420–3,045)	1,446 (436–2,132)	2,469 (727–3,652)
2028	8,911 (6,281–10,696)	2,855 (2,299–3,394)	2,293 (2,092–3,040)	1,392 (424–2,050)	2,371 (709–3,499)
2029	8,439 (5,945–10,132)	2,705 (2,525–3,467)	2,172 (2,024–2,960)	1,317 (399–1,941)	2,245 (670–3,314)
2030	8,014 (5,619–9,637)	2,555 (2,480–3,099)	2,050 (1,989–2,826)	1,268 (380–1,870)	2,141 (635–3,163)
2031	7,905 (5,773–9,350)	2,878 (2,798–3,392)	2,018 (1,962–3,130)	1,324 (388–1,959)	1,685 (489–2,497)
2032	7,510 (5,484–8,883)	2,734 (2,618–3,360)	1,917 (1,836–3,090)	1,258 (369–1,861)	1,601 (465–2,372)
2033	7,114 (5,195–8,415)	2,590 (2,464–3,432)	1,816 (1,729–3,011)	1,191 (350–1,763)	1,517 (440–2,247)
2034	7,099 (5,180–8,400)	2,581 (2,440–3,160)	1,810 (1,712–2,508)	1,191 (350–1,763)	1,517 (440–2,247)
2035	7,084 (5,165–8,385)	2,572 (2,424–2,985)	1,804 (1,701–2,300)	1,191 (350–1,763)	1,517 (440–2,247)
2036	6,683 (4,876–7,909)	2,429 (2,267–2,835)	1,703 (1,591–2,295)	1,119 (328–1,655)	1,432 (416–2,122)
2037	6,669 (4,862–7,895)	2,421 (2,261–2,831)	1,698 (1,587–2,292)	1,118 (328–1,655)	1,432 (416–2,122)
2038	6,566 (4,813–7,754)	2,413 (2,250–2,827)	1,690 (1,579–2,295)	1,143 (330–1,694)	1,320 (380–1,957)
2039	6,532 (4,777–7,721)	2,404 (2,233–2,813)	1,675 (1,562–2,284)	1,140 (325–1,693)	1,313 (373–1,950)
2040	6,519 (4,769–7,706)	2,404 (2,205–2,803)	1,669 (1,533–2,276)	1,140 (325–1,693)	1,306 (371–1,941)
2041	6,219 (4,523–7,370)	2,255 (2,067–2,794)	1,570 (1,443–2,269)	1,046 (307–1,548)	1,348 (391–1,997)
2042	6,053 (4,401–7,172)	2,191 (1,990–2,784)	1,531 (1,392–2,261)	1,020 (299–1,509)	1,311 (381–1,942)
2043	5,814 (4,227–6,889)	2,103 (1,901–2,778)	1,472 (1,329–2,256)	981 (288–1,451)	1,258 (365–1,864)
2044	5,577 (4,055–6,608)	2,016 (1,802–2,775)	1,413 (1,264–2,253)	942 (276–1,393)	1,206 (350–1,787)
2045	5,419 (3,940–6,421)	1,958 (1,722–2,765)	1,374 (1,210–2,245)	916 (269–1,354)	1,171 (340–1,735)
2046	5,337 (3,881–6,324)	1,928 (1,677–2,761)	1,354 (1,179–2,242)	903 (265–1,335)	1,152 (335–1,707)
2047	5,255 (3,821–6,226)	1,897 (1,626–2,755)	1,335 (1,145–2,239)	889 (261–1,316)	1,134 (329–1,679)
2048	5,174 (3,762–6,131)	1,867 (1,574–2,746)	1,315 (1,111–2,237)	876 (257–1,296)	1,116 (324–1,654)
2049	5,010 (3,641–5,939)	1,807 (1,501–2,742)	1,271 (1,061–2,229)	850 (249–1,258)	1,082 (314–1,603)
2050	4,849 (3,524–5,747)	1,751 (1,427–2,738)	1,228 (1,008–2,145)	824 (242–1,219)	1,046 (304–1,549)

Notes: LR, low-risk; HR, high-risk; MSM, men who have sex with men.

The number of new HIV infections and its proportion in the four risk groups from our model varied, as shown in Table 2. The predicted number of the new HIV infections between 2016 and 2050 among heterosexual women was 2,067 (UI: 2,359–3,016) in 2016, which increased to 2,835 (UI: 2,259–2,879) in 2019, jumped to 4,346 (UI: 3,612–4,735) in 2025, and then decreased to 1,228 (UI: 1,008–2,145) in 2050; among heterosexual men, the number was 2,839 (UI: 2,237–3,243) in 2016, which increased to 3,189 (UI: 2,698–3,842) in 2018, and then decreased to 1,751 (UI: 1,427–2,738) in 2050; among high-risk MSM, the number was 2,288 (95% CrI: 660–3,393) in 2016, which slightly increased to 2,568 (95% CrI: 756–3,798) in 2025 and then decreased to 1,046 (95% CrI: 304–1,549) in 2050; and among low-risk MSM, the number was 1,094 (95% CrI: 329–1,612) in 2016, which increased to 1,209 (95% CrI: 357–1,787) in 2019 then to 1,487 (448–2,193) in 2026 and decreased to 824 (95% CrI: 242–1,219) in 2050. The predicted number of new HIV infections can be broken down as follows: women accounted for approximately 25.0% of the total, homosexual transmissions accounted for approximately 40.0% of the total, high-risk MSM accounted for approximately 25.0% of the total, and MSM accounted for 55.0% of men.

## Discussion

Despite including the period of the COVID-19 pandemic in this prediction, the predicted number of new HIV infections was similar to the number during the HIV epidemic in Guangdong based on the relative ratios from 2016 to 2022. As measures and strategies for HIV prevention and control were carried out regularly before the COVID-19 pandemic in 2016 and 2019, the number of new HIV infections also increased slightly, similar to the number of new HIV diagnoses, which showed a changing trend. Overall, the predicted number increased during the COVID-19 pandemic and for approximately two or three years after the pandemic. The proportion of women out of the total predicted number peaked at 39% during this period. Then, the predicted number decreased continually until 2050.

A range of calibrated criteria, the relative ratios, between the predicted number in this model and HIV diagnoses had values larger than 1.2, fitted with the proportions of HIV diagnoses from Guangdong Provincial AIDS expert interviews on World AIDS Day, which was 71.3% in 2019 [58] and 78.7% in 2021 [57], and the interpretations or viewpoints of national experts regarding the predicted results in China evaluated by Spectrum, which was recommended worldwide [3]. The predicted number of new HIV infections jumped during the COVID-19 pandemic, with the calibrated criterion ranging from 1.4 to 1.7 due to the interruption of or delay in HIV services provided by health care workers who had been diverted for COVID-19 [59] to different extents. These services included HIV testing and counselling, referrals, timely antiretroviral treatment, and the promotion in condom use. Previous studies that used simulation models and cross-sectional data reported that the COVID-19 pandemic should have led to an increase in the number of new HIV infections [43, 60]. HIV diagnoses in another province of China declined by approximately 37.0% during the COVID-19 pandemic compared to the same period before the pandemic, according to a prediction model [61]. Furthermore, there were no convincing data to imply that the variety of sexual behaviours in risk groups differed from that before the COVID-19 pandemic [62].

The predicted number of new HIV infections increased continually for two or three years after the end of the COVID-19 pandemic. Recognizing that high-risk individuals may have been unaware that they had HIV due to reductions in HIV testing, there should have been an increase in HIV transmission because those individuals who worried about contracting COVID-19 did not go to designated institutions for HIV testing. As the COVID-19 epidemic was mitigated, HIV testing failed to promptly reduce HIV transmission, but risk behaviours for HIV may have been unchanged and potentially increased [63] when the COVID-19 epidemic was mitigated. Cross-sectional data showed that the HIV-positive rate of blood donors was much higher than that before the COVID-19 pandemic [64]. The predicted new HIV infections and the trend after the COVID-19 pandemic showed brief increases and then steadily declined after measures and strategies for prevention and control were reinstated, especially scaled up HIV testing [65], timely antiretroviral treatment for newly diagnosed infections, and viral suppression.

Although we used proportions of late diagnoses in addition to new HIV diagnoses from 2016 to 2022 in Guangdong Province to calibrate our model, it was and remains uncertain how many new HIV infections there were and how many were diagnosed in China based on existing research and the viewpoints of national or provincial core experts. The diagnosed proportion of new HIV infections was 68.9% (61.5%, 78.3%) [55] based on predictions made in 2018 by Spectrum regarding the HIV epidemic in China. The diagnosis rate of HIV infections remains low, and the HIV epidemic may be increasing continuously, which is in contrast to predictions from the workbook method and core experts indicating that the HIV epidemic has been stable since 2007 in China [3].

Nevertheless, regarding HIV transmission from MSMW to general women [28], the sex compositions and proportions of heterosexual and homosexual sexual acts associated with the number of new HIV infections from our model were similar to those related to the number of new HIV diagnoses from 2016 to 2019 in Guangdong. The female proportion of new HIV diagnoses and the proportion of heterosexual transmission of new HIV diagnoses in Guangdong during recent years were approximately 30.0% and over 60.0% [66], respectively, which were similar to the national proportions, with the proportions were approximately 30.0% and 70.0% among people living with HIV, respectively [3]. The female proportion of the new HIV infections was higher during the COVID-19 epidemic, and after two years, for the proportion of heterosexual acts among MSMW may increase due to a decline of approximately one-fifth in the number of male sexual partners among MSMW in that period [43], which further could lead to increase for heterosexual transmission among MSMW.

The model parameters and population size in our model were selected based on more reliable sources. The ranges of the parameters were set according to highly cited studies and the viewpoints of core experts, and the prior distribution of the mixing index was reasonable. The model parameters of the HIV epidemic in Guangdong in our model were preferable, and they were replaced with parameters from another province with a similar HIV epidemic were replaced if these parameters for Guangdong were not acquired; otherwise, the values from a meta-analysis were used for China. For some parameters, including the effectiveness of condoms, we opted for values from highly cited studies.

Our modelling study had three strengths. First, the assumptions in the model were in line with the characteristics of the HIV epidemic in Guangdong Province and Chinese social norms and pressures on MSM, including low- and high-risk subgroups classified according to the number of sexual partnerships and the higher proportion of heterosexual sexual acts in China [17, 20]. Second, the calibrated criteria in the model also included the proportion of late diagnoses, which was approximately 30.0% and even higher during the COVID-19 epidemic, in addition to the new HIV diagnoses each year in Guangdong Province. Finally, this is the first compartmental model to publicly predict the number of new HIV infections in members of the population with susceptibility to sexual transmission from 2016 to 2050 in Guangdong Province. However, this study also had four limitations. First, our model failed to identify the risk groups of sexual partnerships, MSMW or heterosexual men, among females with HIV infections. The problem could be further explored by defining of subgroups for females in our model. Second, our model failed to subclassify according to CD4 cell counts, considering that viral suppression may be more reasonably defined as a state of being “Treated”, starting in 2016 for all people with HIV initiating antiretroviral treatment regardless of clinical stage and CD4 cell count. Third, the mixing index failed to randomize between low- and high- risk MSM but did randomize between heterosexual and homosexual sexual acts because HIV transmission from MSM to women may be substantial, impacted by Chinese traditional cultures. Finally, the calibration criterion for our model would also lead to an underestimation of new HIV infections, while we have taken into account the proportion of late diagnoses. And determining an upper value for model calibration is challenging due to the lack of available data.

## Conclusion

We developed a deterministic compartmental model to predict the number of new HIV infections from 2016 to 2050. The presumption regarding MSM who engaged in heterosexual acts and the criterion of model calibration may all be in line with the complicated characteristics of the epidemic in Guangdong Province to some degree. The results from the model may simulate a realistic epidemic in Guangdong Province by model calibration. The predicted number slightly increased between 2016 and 2019, was much larger during the COVID-19 pandemic until two years after the end of the pandemic, and then continually declined until 2050. Overall, the HIV epidemic in Guangdong Province remains serious, and it is urgently important to restore services for HIV control and prevention after the COVID-19 pandemic.

### Electronic supplementary material

Below is the link to the electronic supplementary material.

Supplementary Material 1

## Data Availability

No datasets were generated or analysed during the current study.
