# Supplementary material for: New sexually transmitted HIV infections from 2016 to 2050 in Guangdong Province, China: a study based on a dynamic compartmental model
Source: BMC Public Health. 2024 May 14;24:1307. doi: 10.1186/s12889-024-18735-z (PMC11092022; doi:10.1186/s12889-024-18735-z)
Supplement: Supplementary file 1 — Supplementary Material 1 [file 12889_2024_18735_MOESM1_ESM.docx]

**Supplementary material**

We developed a deterministic and dynamic compartmental model of HIV transmission for the entire population that included people in four risk groups aged 15 and older in Guangdong from 2016 to 2050. The model projected the number of new HIV infections and their 95% credible intervals in this population, which were calibrated by data on new HIV diagnoses published on government websites and undiagnosed proportions of HIV infections. We used the Morris and Sobol methods to analyze the sensitivity of the model parameters.

##

$$\frac{X_{j,1,i=1}}{dt}=\zeta_{j,1}\sum_{j=1}^{4} X_{j,1,i}-\left( \sum_{k=2,3,4^{'}} \lambda_{i,k}^{j}\left( t \right) \right)X_{j,1,i}-d_{j,1}X_{j,1,i} i=1 \left( 1 \right)$$

$$\frac{X_{j,2,i=1}}{dt}=\left( \sum_{k=2,3,4^{'}} \lambda_{i,k}^{j}\left( t \right) \right)X_{j,1,i}-d_{j,2}X_{j,2,i} i=1 \left( 2 \right)$$

$$\frac{X_{j,3,i}}{dt}=\delta_{j,2}X_{j,2,i}-d_{j,3}X_{j,3,i} i=1, 2 \left( 3 \right)$$

$$\frac{X_{j,4,i}}{dt}=\psi_{j,3}X_{j,3,i}-d_{j,4}X_{j,4,i} i=1, 2 \left( 4 \right)$$

Population sizes of the risk groups

The number of people in the risk groups, including heterosexual men, heterosexual women, low-risk and high-risk MSM, and low-risk and high-risk MSMW, whose risk groups among MSM or MSMW were classified based on the number of male sexual partners among MSM, were calculated on the basis of the population size and the proportion of people aged 15 and over by sex from then the 2020 Guangdong Statistical Yearbook, which includes data from 2019 and before [1]. Data from 2020 were used with the assumption that the population size has remained relatively steady. Presuming that the population size of low-risk MSM was equal to that of high-risk MSM [2], the proportion of MSM was 5.0% of all men aged 15 and over, and the proportion of heterosexual sexual acts among low- and high-risk MSM was 31.2% [3]. We defined MSM as the high-risk MSM who satisfied at least one of two conditions : 1) more than 10 sexual partners over the past six months [4] and 2) rates of inconsistent condom use over the past six months more than 50.0%, which were approximately 50.0% in Guangdong; otherwise, they were classified as low-risk MSM [2]. By excluding people already living with HIV, we estimated that the population sizes of heterosexual men, low- and high-risk MSM, and low- and high-risk MSMW were 47.89 million, 1.2 million, 1.2 million, 0.2 million, and 0.2 million, respectively. The population size of heterosexual women was 43.73 million, which excluded people living with HIV and a number of lesbians, whose proportion was presumed to be 5.0%, as the risk of HIV transmission is extremely rare in this group.

## Sensitivity analysis

Considering the cost of calculations, we combined a qualitative global method and a quantitative global method, the Morris and Sobol methods, to analyze the sensitivity of model parameters to the number of new HIV infections predicted in our model. The first step qualitatively determined the sensitive parameters via the Morris method [5] and then calculated the quantitative effect of those identified parameters on the number of new HIV infections predicted via the Sobol method [6]. The sensitivity analysis excluded the parameter of the mixing index because it was certainly sensitive. The outputs of the Sobol method included the first-order indices, the second-order indices, and the total-order indices on qualitatively identified parameters from the absolute values of the elementary effects ($\left| EEs \right|$) of the Morris method. Second-order indices are used if the total-order indices are sufficiently larger relative to the first-order indices.

# Variables and definitions

**Supplementary Table 1**

Variables and definitions.

| **parameters** | **Description** | **Values (min**, **max)** | **Source** |
| --- | --- | --- | --- |
| **subscripts** | **description** |  |  |
| $j=1,2,3,4$ | Four risk populations | **1,2,3,4** | **-** |
| $k=1,2,3,4$ | Compartment states, including ART failure | **1,2,3,4,**$\boldsymbol{4}^{\boldsymbol{'}}$ | **-** |
| $i=1,2$ | Routes of sexual transmission, 1 being heterosexual, 2 homosexual | **-** | **-** |
| **Variables** | **definition** |  |  |
| $\boldsymbol{X}_{\boldsymbol{k}}\boldsymbol{, k=1,2,\ldots,4}$ | **Populations of the states** |  |  |
| $X_{1}$ | Susceptible population size | 945,451,90 | [1] |
| $X_{2}$ | Infected population size | 9473 | [1] |
| $X_{3}$ | Diagnosed population size | 7455 | [1] |
| $X_{4}$ | Treated population size | 6173 | [1] |
| **Parameters** | **definition** |  |  |
| $\boldsymbol{\theta}$ | Rate of antiretroviral treatment failure | 0.065 | [7] |
| $\boldsymbol{\zeta}_{\boldsymbol{j,k=1}}$ | Annual entry rates of the Susceptible population | 0.105 | [1] |
| ${dD}_{\boldsymbol{j,k}}$ | Annual death rates |  |  |
| $d_{j,1}$ | Death rate in the Susceptible population | 0.037 | [1] |
| $d_{j,2}$ | Death rate in the Infected population | 0.04 | [8, 9] |
| $d_{j,3}$ | Death rate in the Diagnosed population | 0.04 | [8, 9] |
| $d_{j,4}$ | Death rate in the Treated population | 0.08 | [8, 9] |
| $\boldsymbol{\delta}_{\boldsymbol{j},2}$ | Rates of HIV infections diagnosed in the Infected population | 0.787 | [10] |
| $\boldsymbol{\psi}_{\boldsymbol{j},3}$ | Annual proportions of antiretroviral treatment (ART) for HIV infections in the four risk groups | 0.828 | [10] |
| ${nN}_{\boldsymbol{j,i}}$ | Number of partners per person per year in the four risk groups |  |  |
| $n_{1,1}$ | Number of partners per person per year among men who engage in heterosexual sexual acts only | 6 (4, 9) | [11] |
| $n_{2,1}$ | Number of partners per person per year among women who engage in heterosexual sexual acts only | 12 (7, 19) |  |
| $n_{3,1}$ | Number of female partners per person per year among low-risk MSM | 4 (1, 5) | [12, 13] |
| $n_{3,2}$ | Number of same-sex partners per person per year among low-risk MSM | 6 (3, 9) | [4] |
| $n_{4,1}$ | Number of female partners per person per year among high-risk MSM | 4 (1, 5) | [12-14] |
| $n_{4,2}$ | Number of same sex partners per person per year among high-risk MSM | 13 (7, 18) | [4] |
| ${Mu}_{\boldsymbol{j,i}}$ | Proportions of condom use in the four risk groups |  |  |
| $u_{1,1}$ | Proportion of condom use among men who engage in heterosexual sexual acts only | 0.23 (0.13, 0.42) | [15, 16] |
| $u_{2,1}=u_{1,1}$ | Proportion of condom use among women who engage in heterosexual sexual acts only | 0.23 (0.13, 0.42) | [15, 16] |
| $u_{3,1}$ | Proportion of condom use in heterosexual sexual acts among low-risk MSM | 0.23 (0.13, 0.42) | [15] |
| $u_{3,2}$ | Proportion of condom use in homosexual sexual acts among low-risk MSM | 0.50 (0.4， 0.55) | [17, 18] |
| $u_{4,1}$ | Proportion of condom use in heterosexual sexual acts among high-risk MSM | 0.23 (0.13, 0.42) | [15] |
| $u_{4,2}$ | Proportion of condom use in homosexual sexual acts among high-risk MSM | 0.50 (0.4， 0.55) | [17, 18] |
| ${Ee}_{\boldsymbol{i}}$ | Effectiveness of condoms in sexual acts |  |  |
| $e_{1}$ | Effectiveness of condoms in heterosexual sexual acts | 0.8 | [19] |
| $e_{2}$ | Effectiveness of condoms in homosexual sexual acts | 0.7 | [20] |
| ${\pi\Pi}_{jk\boldsymbol{,i}}$ | Susceptible population’s probabilities of acquiring HIV per partnership from the I, D, and ART failure subpopulations (*k* = 2, 3, 4’) in the Susceptible population from unprotected or high-risk sexual acts |  |  |
| $\pi_{1,2/3/4^{'},1}$ | Probability of acquiring HIV among men who engage in heterosexual sexual acts | 0.0006 | [13] |
| $\pi_{22/3/4^{'},1}$ | Probability of acquiring HIV among women who engage in heterosexual sexual acts only | 0.001 | [13] |
| $\pi_{3/42/3/4^{'},2}$ | Probability of acquiring HIV among high-risk MSM | 0.02 | [13] |
| $\boldsymbol{\varepsilon}$ | Mixing index: a randomized level of MSM opting for male or female sexual partners | 0.504 (0.239, 0.854) | Estimation (ABC-SMC) |

# Probability of HIV acquisition

Probability of HIV acquisition was a function of probabilities of susceptible individuals not becoming infected with HIV in sexual acts, the proportions of sexual acts, proportions of condom use and the effectiveness of condoms. The formulas were as follows, including the number of total sexual partners per year in the four risk groups, proportions of sexual acts per year, probabilities of susceptible individuals not becoming infected with HIV in sexual acts, and the probability of HIV acquisition.

## Number of total sexual partnerships

The number of total sexual partnerships per year for the four risk groups ($j=1, 2, 3, 4$) was ${TP}_{j}$. *k* represents the four states of HIV transmission in our model, and *i* represents the route of sexual transmission, including the heterosexual and homosexual routes.

${TP}_{1}=\sum_{k=1}^{4} X_{1,k}n_{1,i=1}$, ${TP}_{2}=\sum_{k=1}^{4} X_{2,k}n_{2,i=1}$,

${TP}_{3}=\sum_{k=1}^{4} \sum_{i=1}^{2} X_{3,k}n_{3,i}$, ${TP}_{4}=\sum_{k=1}^{4} \sum_{i=1}^{2} X_{4,k}n_{4,i}$

## Proportions of sexual acts

The proportions of sexual acts in the four risk groups, $p_{j,i}$, were as follows. $\varepsilon$ denotes the mixing index, ranging from 0 to 1, where 0 denotes that MSM choose male sexual partners and 1 denotes that MSM choose male and female sexual partners completely at random. Heterosexual men and women engaged only heterosexual sexual acts.

$p_{1,1}=\frac{{TP}_{2}-{TP}_{3,i=1}-{TP}_{4,i=1}}{{TP}_{1}+{TP}_{2}-{TP}_{3,i=1}-{TP}_{4,i=1}}$ , $p_{2,1}=\frac{{TP}_{1}+{TP}_{3,i=1}+{TP}_{4,i=1}}{{TP}_{1}+{TP}_{2}+{TP}_{3}+{TP}_{4}}$ ,

$p_{3,1}=\varepsilon\frac{{TP}_{2}-{TP}_{1}}{{TP}_{2}+{TP}_{3}+{TP}_{4}-{TP}_{1}}$ , $p_{3,2}=\left( 1-\varepsilon\right)+\varepsilon\frac{{TP}_{3}+{TP}_{4}}{{TP}_{2}+{TP}_{3}+{TP}_{4}-{TP}_{1}}$ ,

$p_{4,1}=p_{3,1}$ , $p_{4,2}=p_{3,2}\left( 1-\varepsilon\right)+\varepsilon\frac{{{TP}_{3}+TP}_{4}}{{TP}_{2}+{TP}_{3}+{TP}_{4}}$

## Probabilities of susceptible individuals not becoming infected with HIV in risky sexual acts

The probabilities of susceptible individuals not acquiring HIV in unprotected or high-risk sexual acts with HIV infections (*k* = 2,3,4’) per year were ${NP}_{j,i,k=2,3,4^{'}}$. $4^{'}$ denotes the state of ART failure. $X_{j,i}$ denotes population size of the four states of HIV transmission in our model. $\pi_{j,i}$ denotes the probabilities of the susceptible population acquiring HIV from unprotected or high-risk sexual acts among the three other risk groups.

${NP}_{1,1,k=2,3,4^{'}}$ ${NP}_{1,1,2}=\left( 1-\frac{X_{1,21}n_{1,1}}{{TP}_{1}}\pi_{21,1} \right)$

$${NP}_{1,1,4^{'}}=\left( 1-\frac{X_{1,4}{\theta n}_{1,1}}{{TP}_{1}}\pi_{4^{'},1} \right)$$

${NP}_{2,1,k=2,3,4^{'}}$

${NP}_{2,1,2}=\left( 1-\frac{X_{2,2}n_{2,1}}{{TP}_{2}}\pi_{2,1} \right)$ ${NP}_{2,1,4^{'}}=\left( 1-\frac{X_{2,4}{\theta n}_{2,1}}{{TP}_{2}}\pi_{4^{'},1} \right)$ ${NP}_{3,i,k=2,3,4^{'}}$ ${NP}_{3,1,2}=\left( 1-\frac{X_{3,2}n_{3,1}}{{TP}_{3}}\pi_{2,1} \right)$

${NP}_{3,2,2}=\left( 1-\frac{X_{3,2}n_{3,2}}{{TP}_{3}}\pi_{3/42,2} \right)$ ${NP}_{3,1,3}=\left( 1-\frac{X_{3,3}n_{3,1}}{{TP}_{3}}\pi_{3,1} \right)$ ${NP}_{3,1,4^{'}}=\left( 1-\frac{X_{3,4}\theta n_{3,1}}{{TP}_{3}}\pi_{4^{'},1} \right)$ ${NP}_{4,i,k=2,3,4^{'}}$ ${NP}_{4,1,2}=\left( 1-\frac{X_{4,2}n_{4,1}}{{TP}_{4}}\pi_{2,1} \right)$

${NP}_{4,2,2}=\left( 1-\frac{X_{4,2}n_{4,2}}{{TP}_{4}}\pi_{3/42,2} \right)$ ${NP}_{4,1,3}=\left( 1-\frac{X_{4,3}n_{4,1}}{{TP}_{4}}\pi_{3,1} \right)$ ${NP}_{4,1,4^{'}}=\left( 1-\frac{X_{4,4}{\theta n}_{4,1}}{{TP}_{4}}\pi_{4^{'},1} \right)$

## Probability of HIV acquisition

The formula to indirectly calculate via NP the probability of HIV acquisition was as follows.

$$\sum_{k=2,3,4^{'}} \lambda_{i,k}^{j}\left( t \right)=\sum_{k=2,3,4^{'}} \left\{ 1-{{NP_{Hetero_{male}}}_{1,k}}^{p_{1,1}n_{1,1}\left( 1-u_{1,1}e_{i1} \right)} \right\}+\sum_{k=2,3,4^{'}} \left\{ 1-{{NP_{Hetero_{female}}}_{1,k}}^{p_{2,1}n_{2,i1}\left( 1-u_{2,1i}e_{i1} \right)} \right\}+\sum_{k=2,3,4^{'}} \left\{ 1{{-NP_{{LR}_{Homo}}}_{2i,k}}^{p_{3,i2}n_{3,i2}\left( 1-u_{3,i2}e_{i2} \right)} \right\}+\sum_{k=2,3,4^{'}} \left\{ 1{{-NP_{{HR}_{Homo}}}_{2i,k}}^{p_{4,i2}n_{4,i2}\left( 1-u_{4,i2}e_{i2} \right)} \right\}$$

# Results

Parameter Estimation

The value of the mixing index estimated from ABC-SMC was 0.504 (95% CrI: 0.239, 0.894). The prior presumed distribution of the index was uniformly distributed [0, 1], the sampling sequences were set at 25, the total sample size of each sequence was 10,000, each sampling size was 10, and the effective sample size was also estimated.

Sensitivity Analysis

The $\left| EEs \right|$ from the Morris method for the number of sexual partners, probabilities of acquiring HIV during high-risk sexual acts, the proportion of condom use and the population sizes of the four states were 3,811.9, 2,441.1, 136.0 and 440.4, respectively. The first three parameters were identified as sensitive parameters, as the population sizes were relatively stable. The Sobol method revealed the following ranking for the three parameters: the number of sexual partners, the probability of acquiring HIV and the proportion of condom use (see the Supplementary Figure 2). The total indices were calculated to be 0.73, 0.29 and 0.0009, respectively, while the first-order indices were found to be 0.70, 0.27, and 0.0007, respectively.


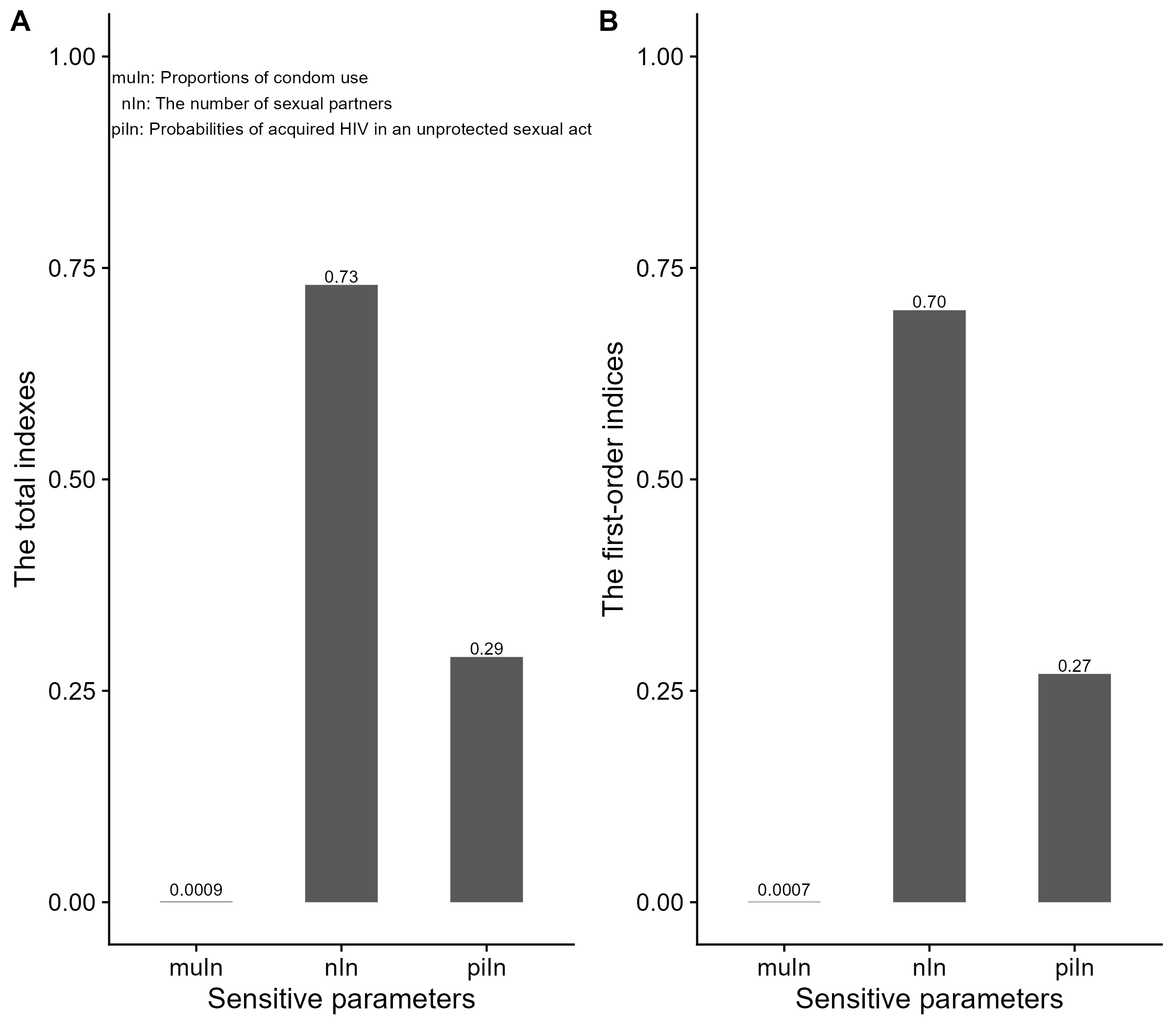


**Supplementary Fig. 2.** The results of the Sobol sensitivity indices in our compartmental model.

# References

1. Statistics Bureau of Guangdong Province, Survey office of Ntional Bureau of Statistics in Guangdong. 2020 Guangdong statistical yearbook: 2019 permanent population of Guangdong. Beijing: China Statistics Press; 2020.

2. Fu X, Lin P, Wang Y, Li J, Yu G, Li Y. Prevalence of human immunodeficiency virus and syphilis among MSM in Guangdong from 2009 to 2013. *Chin Prev Med*. 2014; 15(5):169-172.

3. Yun K, Xu JJ, Reilly KH, Zhang J, Jiang YJ, Wang N, Shang H. Prevalence of bisexual behaviour among bridge population of men who have sex with men in China: a meta-analysis of observational studies. *Sex Transm Infect*. 2011; 87(7):563-570.

4. Zhang L, Chow EP, Wilson DP. Distributions and trends in sexual behaviors and HIV incidence among men who have sex with men in China. *BMC Public Health*. 2012; 12:546.

5. Campolongo F, Cariboni J, Saltelli A. An effective screening design for sensitivity analysis of large models. *Environ Model Softw*. 2007; 22(10):1509-1518.

6. Saltelli A, Annoni P, Azzini I, Campolongo F, Ratto M, Tarantola S. Variance based sensitivity analysis of model output. Design and estimator for the total sensitivity index. *Comput Phys Commun*. 2010; 181(2):259-270.

7. He N. Emerging changes and characteristics of the HIV epidemic in China. *Shanghai Preventive Medicine*. 2019; 31(12):963-967.

8. Cao W, Hsieh E, Li T. Optimizing treatment for adults with HIV/AIDS in China: Successes over two decades and remaining challenges. *Curr HIV/AIDS Rep*. 2020; 17:26-34.

9. Li CB, Zhou Y, Wang Y, Liu S, Wang W, Lu X, Sun CM, Liu P, Hu Q-H, Wen Y. In-hospital Mortality and Causes of Death in People Diagnosed With HIV in a General Hospital in Shenyang, China: A Cross-Sectional Study. *Frontiers in Public Health*. 2021; 9.

10. Zhang H. Sexual transmission as a dominant route and new diagnoses increasing among older people. Guangzhou: YangCheng Evening News. 2021. <https://baijiahao.baidu.com/s?id=1717928090025091240&wfr=spider&for=pc>.

11. Wu S, Wang L, Chen Y, Xu L. [Status of HIV and STD infection in a STD clinic in Shaoguan city in 2014-2018]. *J Trop Med*. 2019; 19(12):1557-1560.

12. Cai R, Zhao J, Cai W, Chen L, Richardus JH, de Vlas SJ. HIV risk and prevention behaviors in men who have sex with men and women: a respondent-driven sampling study in Shenzhen, China. *AIDS Behav*. 2014; 18(8):1560-1568.

13. Wu X, Dou Q, Bao Y, Zhang Y, Tan H. Characteristics of HIV's sexual behavior and their effect on the secondary transmission rate. *J Cent South Univ (Med Sci)*. 2016; 41(7):715-720.

14. Ha TH, Liu H, Liu H, Cai Y, Feng T. Concurrent sexual partnerships among men who have sex with men in Shenzhen, China. *Sex Transm Dis*. 2010; 37(8):506-511.

15. Chow EP, Wilson DP, Zhang L. What is the potential for bisexual men in China to act as a bridge of HIV transmission to the female population? Behavioural evidence from a systematic review and meta-analysis. *BMC Infect Dis*. 2011; 11:242.

16. Han J, Guo J, Yan J. Sexual behavior characteristics and condom use of newly discovered HIV/ AIDS cases with heterosexual transmission in Zhumadian of Henan. *Henan J Prev Med*. 2021; 32(6):467-470, 477.

17. He Q, Wang Y, Lin P, Liu Y, Yang F, Fu X, Li Y, Sun B, Li J, Zhao X*, et al*. Potential bridges for HIV infection to men who have sex with men in Guangzhou, China. *AIDS Behav*. 2006; 10(Suppl 4):S17-23.

18. Huang Q, Li Q, Li Y, Zeng G, Cui X, Yan P, Lyu F, Luan R. Prevalence of HIV infection and syphilis, sexual behaviors and awareness of HIV/AIDS related knowledge among men who have sex with men in China: a Meta-analysis of data collected from 2010 to 2013. *Zhonghua Liu Xing Bing Xue Za Zhi*. 2015; 36(11):1297-1304.

19. Weller SC, Davis-Beaty K. Condom effectiveness in reducing heterosexual HIV transmission. *Cochrane Database Syst Rev*. 2002(1):CD003255.

20. Smith DK, Herbst JH, Zhang X, Rose CE. Condom effectiveness for HIV prevention by consistency of use among men who have sex with men in the United States. *J Acquir Immune Defic Syndr*. 2015; 68(3):337-344.
